# Pre- and post-headache phases of migraine: multi-country results from the CaMEO – International Study

**DOI:** 10.1186/s10194-023-01683-1

**Published:** 2023-11-08

**Authors:** Richard B. Lipton, Michel Lanteri-Minet, Elizabeth Leroux, Aubrey Manack Adams, Janette Contreras-De Lama, Michael L. Reed, Kristina M. Fanning, Dawn C. Buse

**Affiliations:** 1 Albert Einstein College of Medicine, Bronx, NY USA; 2 Pain Department and FHU InovPain, CHU Nice and Côte Azur University, Nice, France; 3 INSERM U1107 Migraine and Trigeminal Pain, Auvergne University, Clermont-Ferrand, France; 4 Brunswick Medical Center, Montreal, QC Canada; 5 AbbVie, 2525 Dupont Dr, Irvine, CA 92612 USA; 6 Vedanta Research, Chapel Hill, NC USA; 7 MIST Research, Wilmington, NC USA

**Keywords:** Migraine, Disease burden, Neck pain, Photophobia, Fatigue, Headache disorders, Health surveys, Patient reported outcome measures

## Abstract

**Background:**

Individuals with migraine frequently experience pre- and post-headache symptoms. This analysis aimed to characterize the relative frequency and burden of pre- and post-headache symptoms in people with migraine using data collected through the Chronic Migraine Epidemiology and Outcomes – International Study.

**Methods:**

This cross-sectional, observational, web-based survey was conducted in 2021–2022 in Canada, France, Germany, Japan, the United Kingdom, and the United States. Respondents who met modified *International Classification of Headache Disorders*, 3rd edition, criteria were offered the opportunity to participate. Information collected included migraine-related disability, depression/anxiety symptoms, cutaneous allodynia, activity limitations, and acute treatment optimization. Respondents indicated how often they had pre- or post-headache symptoms using a 5-point scale, ranging from 0 to 4, with a rating of 2 or higher classified as a pre- or post-headache symptom case. Modeling was used to examine relationships with monthly headache days (MHDs) and activity limitations during pre-headache and post-headache phases.

**Results:**

Among a total of 14,492 respondents, pre-headache symptoms were reported by 66.9%, while post-headache symptoms were reported by 60.2%. Both pre-headache and post-headache symptoms were reported by 49.5% of respondents, only pre-headache by 17.4%, only post-headache by 10.7%, and neither pre- nor post-headache symptoms by 22.4%. Compared with respondents who experienced only pre- or post-headache symptoms, respondents who experienced both pre- and post-headache symptoms had the highest rates of 4–7, 8–14, and ≥ 15 monthly headache days (23.1%, 14.1%, and 10.9%, respectively). Of respondents with both pre- and post-headache symptoms, 58.5% reported moderate-to-severe disability, 47.7% reported clinically significant symptoms of depression, 49.0% reported clinically significant symptoms of anxiety, and 63.8% reported cutaneous allodynia with headache (ASC-12). Moderate-to-severe activity limitations were reported during the pre-headache (29.5%) and post-headache phases (27.2%). For all outcomes modeled, after controlling for covariates, having pre-headache symptoms, post-headache symptoms, or both were associated with worse outcomes than having neither.

**Conclusions:**

Pre- and post-headache phases of migraine are common, carry unrecognized burden, and may be a target for treatment.

**Graphical Abstract:**

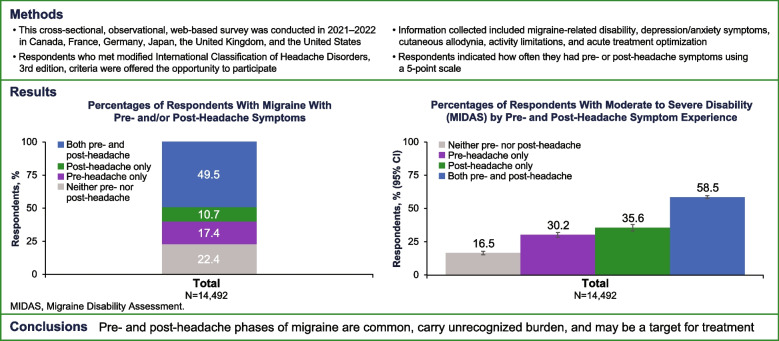

## Introduction

Migraine is a debilitating chronic disease with episodic attacks. Phases of migraine attacks can include prodrome, aura, headache, postdrome, and also an interictal period (between migraine attacks) [1, 2]. Additional research is needed to better understand the pre-headache and post-headache phases of migraine attacks in terms of symptomology and pathophysiology to inform targeted treatments for symptoms that occur before or after the headache phase of an attack [1, 3–5].

Pre- and post-headache symptoms are commonly experienced by people with migraine [6, 7]. The pre-headache phase can include the prodrome and aura phases. The prodrome phase is characterized by symptoms that may begin hours to days before the headache and may include neck stiffness, fatigue, photophobia, and phonophobia, among other symptoms [2, 6, 8, 9]. Aura symptoms may include visual, sensory, speech and/or language, and motor disturbances. According to the *International Classification of Headache Disorders*, 3rd edition (ICHD-3), criteria, aura symptoms spread slowly over a period of 5–60 min before headache pain begins. Post-headache, or postdromal, symptoms can last up to 48 h after the resolution of the headache and may include difficulty concentrating, feeling tired, and neck stiffness, among other symptoms [2]. Symptoms can vary both among individuals and within individuals across attacks.

Responses from the Migraine Clinical Outcome Assessment System (MiCOAS) study’s qualitative interviews of 40 participants with migraine demonstrate a range of both defining cardinal migraine symptoms (e.g., photophobia, phonophobia) and non-cardinal symptoms (e.g., neck tension, vertigo, fatigue/exhaustion) that can be experienced during almost all phases of the migraine cycle and can be both bothersome and impactful [10, 11]. Further analysis of the frequency and types of symptoms reported during migraine phases showed that many of the migraine-defining symptoms occurred during the pre-headache and headache phases; however, some participants noted that once increasing head pain occurs during the headache phase, they attended less to other symptoms [12]. Additionally, a range of emotional/psychological symptoms were reported during the migraine phases, with the pre-headache phase commonly encompassing anxiety, and irritability/impatience, and the post-headache phase sometimes including feelings of relief and euphoria [13].

In a questionnaire study that included 461 participants, 86.9% of participants reported experiencing at least 1 pre-headache symptom and 71.1% reported experiencing at least 2 symptoms [14]. Additionally, a cross-sectional study showed an association between greater headache frequency, duration, and headache pain intensity and a higher number of pre-headache symptoms [6]. In an electronic diary study of 120 participants, more than 80% reported at least 1 post-headache symptom, which may have contributed to the migraine-related disability experienced by the participants [4]. As described already, people with migraine have symptoms beyond the headache phase of a migraine attack, and a better understanding of the frequency and burden of these pre- and post-headache symptoms is needed to better treat people with migraine.

The Chronic Migraine Epidemiology and Outcomes – International (CaMEO-I) Study sought to assess demographics, rates of migraine diagnosis, and treatment patterns among people who met criteria for migraine across 6 countries [15]. The objective of this analysis was to characterize the relative frequency of and burden associated with pre- and post-headache symptoms in people with migraine using data collected through the CaMEO-I Study.

## Methods

### Study design

Methods for CaMEO-I have previously been described [15]. CaMEO-I was a cross-sectional, observational, web-based survey conducted in 2021–2022 in North America (Canada, United States), Europe (France, Germany, United Kingdom), and Asia (Japan). Respondents who met modified ICHD-3 (mICHD-3) migraine symptom criteria were offered the opportunity to participate. Pre- and post-headache symptoms were assessed by evaluating specific items selected from a standardized questionnaire designed by migraine experts. The conduct of the study was governed by the Declaration of Helsinki and its amendments, and any applicable national guidelines. All study participants were required to provide informed consent prior to enrollment in the study.

### Assessments

Respondents answered the questions “Over the past 12 months, how often have you had any premonitory or prodromal symptoms (such as neck pain or stiffness, difficulty thinking, feeling tired, irritable, lightheaded, yawning, vision problems, etc) within a day or two before the start of your migraine or severe headache pain?” and “After the headache pain ends, how often in the past 12 months did you experience a post-headache phase with “post-dromal” symptoms such as fatigue, mental slowness, body aches, and continued sensitivity to light or sound, etc?” using a 5-point scale: 0–never, 1–rarely, 2–less than half the time, 3–half the time or more (but not every time), and 4–with all or almost every headache. Respondents who reported symptoms with a rating of 2 or higher were classified as pre- or post-headache symptom cases for the analysis.

Monthly headache days (MHDs), migraine-associated disability (using the Migraine Disability Assessment [MIDAS]), clinically significant depression and anxiety symptoms (using the Patient Health Questionnaire-4 [PHQ-4]), the presence of cutaneous allodynia with headache (using the Allodynia Symptom Checklist [ASC-12]), and activity limitations due to migraine were assessed. MHDs were determined by responses to the question: “How many days did you have a headache in the past 30 days?” MIDAS is a 5-item questionnaire that evaluates days of missed activity or substantially reduced activity due to headache, including productivity at work or school, household work, and family, social, or leisure activities [16]. Based on the sum score of responses to the items, migraine-related disability was categorized as little or no disability (Grade I), mild disability (Grade II), moderate disability (Grade III), or severe disability (Grade IV). The PHQ-4 is a validated 4-item questionnaire that assesses how often respondents experienced clinically significant levels of depression symptoms (i.e., little interest or pleasure in doing things and feeling down, depressed or hopeless) and/or anxiety symptoms (i.e., feeling nervous, anxious, or on edge and not being able to stop or control worrying) over the previous 2 weeks [17]. PHQ-4 total scores ranged from 0 to 12. A score of ≥ 3 for depression or anxiety symptoms suggest the presence of depression or anxiety, respectively. The ASC-12 is a validated 12-item assessment that evaluates how often respondents experience increased pain or an unpleasant sensation on their skin during their migraine or severe headaches when they engage in various activities of daily living (e.g., combing hair, shaving face, wearing eyeglasses, taking a shower, being exposed to heat or cold) [18]. Based on response options of never, rarely, less than half the time, and half the time or more, total score ranges from 0 to 24. Respondents with an ASC-12 score of ≥ 3 were classified as having allodynia. To assess functional disability due to pre-headache, headache, and post-headache symptoms (assessed separately), respondents were asked to rate their performance of daily activities when they had these symptoms with the following response options: no disability, able to function normally; mildly impaired, can still do everything but with difficulty; moderately impaired, unable to do some things; and severely impaired, cannot do all or most things, bed rest may be necessary.

Among respondents who reported using acute medication for Migraine, acute treatment optimization was assessed using the Migraine Treatment Optimization Questionnaire (mTOQ). The mTOQ-6 is a 6-item questionnaire that evaluates the respondent’s perception of their acute treatment regimen by assessing efficacy at 2 and 24 h, tolerability, ability to plan daily activities, feeling of being in control, and ability to return to normal activities based on response options of never, rarely, less than half the time, and half the time or more [19]. A 4-item subset of the mTOQ-6 was used, referred to herein as the mTOQ-4. The sum of the items was used to categorize treatment optimization as very poor, poor, moderate, or maximal.

Respondents were grouped into the following categories based on their response patterns: with pre-headache symptoms only, with post-headache symptoms only, with both pre- and post-headache symptoms, and without pre- and post-headache symptoms.

### Statistical methods

Descriptive statistics were used in this observational study. Means and standard deviations were generated for age and body mass index. Counts and percentages were used to describe all other variables.

Regression modeling was used to highlight the unique and combined effects of having pre-headache, post-headache, or both types of symptoms on a series of outcomes. Model type reflected the type and distribution of the outcome. MHD categories were modeled with ordinal logistic regression reported with ordered cumulative odds ratios (ORs) and 95% confidence intervals (95% CIs); MIDAS was modeled with a negative binomial regression and reported using rate ratios (RRs) and 95% CIs; the anxiety and depression subscales of the PHQ-4 were modeled with binary logistic regression and reported with ORs and 95% CIs. Very poor to poor vs. moderate to maximum treatment optimization, evaluated via the mTOQ-4, was modeled with binary logistic regression and reported with ORs and 95% CIs. For each outcome (dependent variables), 3 sets of regression models were run: pre-headache only vs. neither, post-headache only vs. neither, and both pre- and post-headache vs. neither as independent variables. Covariates (MHDs, age, gender, country) were added after initial models including only dependent variables and independent variables were run.

Missing data were rare (less than 1% of cases for any variable), and no imputation measures were employed. All analyses were conducted with SPSS Statistics, version 29.0 (IBM, Armonk, NY, USA).

## Results

### Study population

**Fig. 1:**
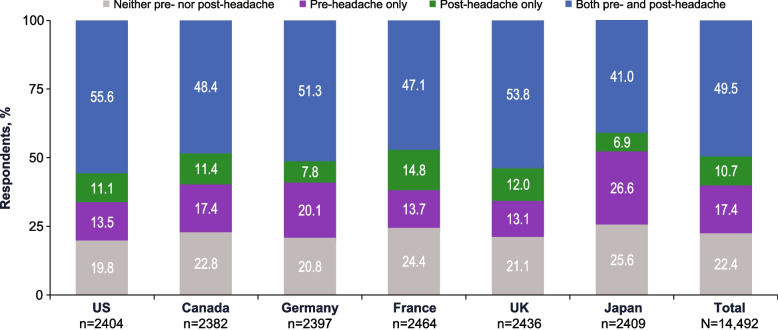
Percentages of Respondents With Migraine With Pre- and/or Post-Headache Symptoms.^a^ Percentages for the overall pre- and/or post-headache symptoms per country were based on the number of respondents who reported typically experiencing symptoms in either or both of the time periods out of the respective migraine sample. UK, United Kingdom; US, United States. ^a^ Includes respondents who reported symptoms “less than half the time,” “half the time or more,” or “with all or almost every headache.”

A total of 14,492 respondents were included in this study (United States: 2404; Canada: 2382; Germany: 2397; France: 2464; United Kingdom: 2436; Japan: 2409). Overall, 17.4% of respondents had pre-headache symptoms only, 10.7% had post-headache symptoms only, 49.5% had both pre- and post-headache symptoms, and 22.4% had neither pre- nor post-headache symptoms (Fig. 1). Demographics for those who reported pre-headache symptoms only, post-headache symptoms only, both pre- and post-headache symptoms, and neither pre- nor post-headache symptoms are presented in Table 1.

**Table 1:** Baseline demographics and characteristics

	**US** ***N=*2404**				**Canada** ***N=*2382**				**Germany** ***N=*2397**				**France** ***N=*2464**			
	Neither *N=*475	Pre Only *N=*325	Post Only *N=*267	Both *N=*1337	Neither *N=*544	Pre Only *N=*415	Post Only *N=*271	Both *N=*1152	Neither *N=*498	Pre Only *N=*481	Post Only *N=*188	Both *N=*1230	Neither *N=*602	Pre Only *N=*337	Post Only *N=*364	Both *N=*1161
**Age, mean (SD), y**	45.3 (15.3)	42.3 (15.0)	41.7 (14.7)	41.3 (13.0)	43.7 (15.4)	42.8 (14.8)	42.4 (15.3)	41.5 (14.5)	43.8 (14.8)	42.5 (15.2)	40.1 (15.8)	40.3 (14.2)	43.5 (15.1)	41.8 (14.3)	40.7 (14.7)	40.8 (13.9)
**Female, n (%)**	284 (59.8)	213 (65.5)	185 (69.3)	970 (72.6)	343 (63.1)	290 (69.9)	208 (76.8)	874 (75.9)	315 (63.3)	336 (69.9)	121 (64.4)	906 (73.7)	404 (67.1)	232 (68.8)	276 (75.8)	882 (76.0)
**BMI, mean (SD), kg/m**^**2**^	27.4 (7.6)	27.8 (7.8)	28.5 (7.7)	29.1 (8.8)	27.2 (7.4)	27.4 (8.2)	27.9 (8.3)	28.3 (8.2)	26.5 (6.7)	26.5 (7.0)	26.1 (6.3)	27.2 (7.6)	25.2 (5.8)	25.1 (6.1)	25.2 (6.6)	25.5 (6.4)
**Employed,**^**a**^** n (%)**	273 (57.5)	187 (57.5)	165 (61.8)	761 (56.9)	327 (60.1)	259 (62.4)	180 (66.4)	662 (57.5)	330 (66.3)	315 (65.5)	119 (63.3)	785 (63.8)	391 (65.0)	226 (67.1)	242 (66.5)	755 (65.0)
**University degree or higher, n (%)**	*N=*475 218 (45.9)	*N=*325 127 (39.1)	*N=*266 114 (42.9)	*N=*1335 424 (31.8)	*N=*541 267 (49.4)	*N=*413 158 (38.3)	*N=*270 123 (45.6)	*N=*1146 430 (37.5)	*N=*490 128 (26.1)	*N=*477 97 (20.3)	*N=*187 40 (21.4)	*N=*1206 237 (19.7)	*N=*599 203 (33.9)	*N=*335 119 (35.5)	*N=*361 122 (33.8)	*N=*1151 343 (29.8)

	**UK** ***N=*2436**				**Japan** ***N=*2409**				**Total** ***N=*14,492**			
	Neither *N=*513	Pre Only *N=*320	Post Only *N=*293	Both *N=*1310	Neither *N=*616	Pre Only *N=*640	Post Only *N=*166	Both *N=*987	Neither *N=*3248	Pre Only *N=*2518	Post Only *N=*1549	Both *N=*7177
**Age, mean (SD), y**	44.1 (15.6)	41.4 (14.6)	43.8 (15.5)	41.1 (13.9)	40.1 (12.7)	41.2 (13.6)	37.0 (11.9)	40.5 (12.8)	43.3 (14.9)	42.0 (14.5)	41.3 (14.9)	41.0 (13.8)
**Female, n (%)**	327 (63.7)	229 (71.6)	212 (72.4)	934 (71.3)	459 (74.5)	487 (76.1)	128 (77.1)	703 (71.2)	2132 (65.6)	1787 (71.0)	1130 (73.0)	5269 (73.4)
**BMI, mean (SD), kg/m**^**2**^	26.9 (8.5)	27.0 (7.5)	27.0 (6.6)	27.4 (8.5)	22.3 (6.1)	21.9 (4.2)	22.4 (5.1)	22.1 (4.6)	25.8 (7.3)	25.5 (7.1)	26.4 (7.2)	26.8 (7.9)
**Employed,**^**a**^** n (%)**	314 (61.2)	203 (63.4)	177 (60.4)	795 (60.7)	380 (61.7)	391 (61.1)	109 (65.7)	658 (66.7)	2015 (62.0)	1581 (62.8)	992 (64.0)	4416 (61.5)
**University degree or higher, n (%)**	*N=*509 220 (43.2)	*N=*318 123 (38.7)	*N=*293 136 (46.4)	*N=*1302 527 (40.5)	*N=*606 289 (47.7)	*N=*636 293 (46.1)	*N=*165 78 (47.3)	*N=*975 454 (46.6)	*N=*3220 1325 (41.1)	*N=*2504 917 (36.6)	*N=*1542 613 (39.8)	*N=*7115 2415 (33.9)

*BMI *Body mass index, *SD *Standard deviation

^a^Full-time, part-time, or self-employed

#### MHD category

**Fig. 2:**
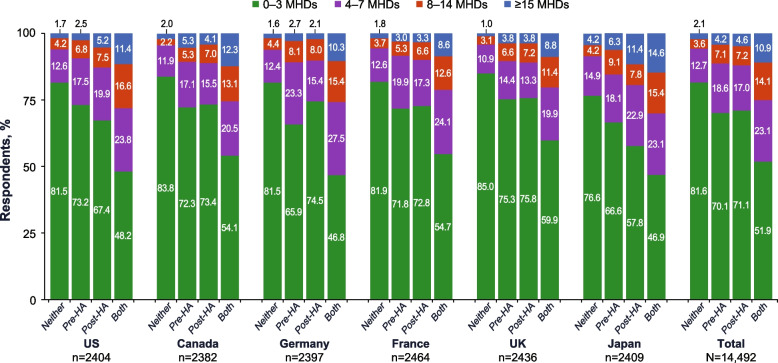
Percentages of Respondents With Each MHD Category by Pre- and Post-Headache Symptom Experience. HA, headache; MHD, monthly headache day

Compared with those with neither type of symptoms, those with pre- or post-headache symptoms or both had more monthly headache days. Of those respondents who had neither pre- nor post-headache symptoms, 12.7% reported 4–7 MHDs, 3.6% reported 8–14 MHDs, and 2.1% reported ≥ 15 MHDs (Fig. 2). Those who experienced only pre- or post-headache symptoms reported higher rates of 4–7 MHDs (pre-headache, 18.6%; post-headache, 17.0%; neither, 12.7%), 8–14 MHDs (pre-headache, 7.1%; post-headache, 7.2%; neither, 3.6%), and ≥ 15 MHDs (pre-headache, 4.2%; post-headache, 4.6%; neither, 2.1%) than those who experienced neither. Respondents who experienced both pre- and post-headache symptoms experienced the highest rates of 4–7 MHDs (23.1%), 8–14 MHDs (14.1%), and ≥ 15 MHDs (10.9%). Based on models adjusted for covariates, those with pre-headache symptoms only, post-headache symptoms only, or both pre- and post-headache symptoms were, respectively, 1.86, 1.94, and 4.29 times more likely to be in a higher MHD category than those with neither type of symptoms.

### Migraine-related burden

#### MIDAS

**Fig. 3:**
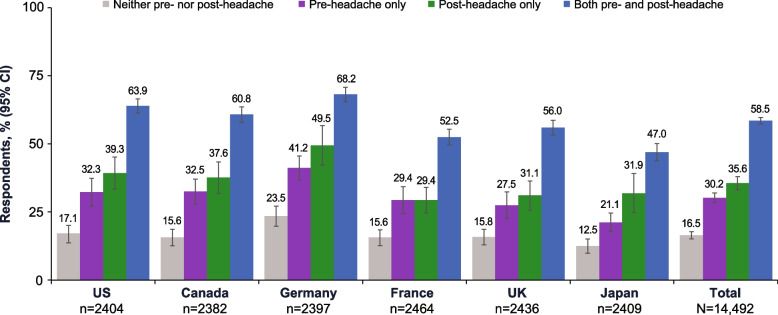
Percentages of Respondents With Moderate-to-Severe Disability (MIDAS) by Pre- and Post-Headache Symptom Experience. MIDAS, Migraine Disability Assessment; UK, United Kingdom; US, United States

Of respondents with neither pre- nor post-headache symptoms, 16.5% scored as having moderate-to-severe disability on the MIDAS (Fig. 3). Moderate-to-severe disability occurred in 51.2% and 54.5% of respondents with any pre-headache or any post-headache symptoms, respectively. Among those with pre-headache symptoms only, 30.2% scored as having moderate-to-severe disability, and among respondents with post-headache symptoms only, 35.6% scored as having moderate-to-severe disability on the MIDAS. Of respondents with both pre- and post-headache symptoms, 58.5% scored as having moderate-to-severe disability on the MIDAS. Based on models adjusted for covariates, having pre-headache symptoms only, post-headache symptoms only, or both pre- and post-headache symptoms increased the risk of disability by 1.78, 1.94, and 3.08 times, respectively, compared with those with neither.

#### PHQ-4

**Fig. 4:**
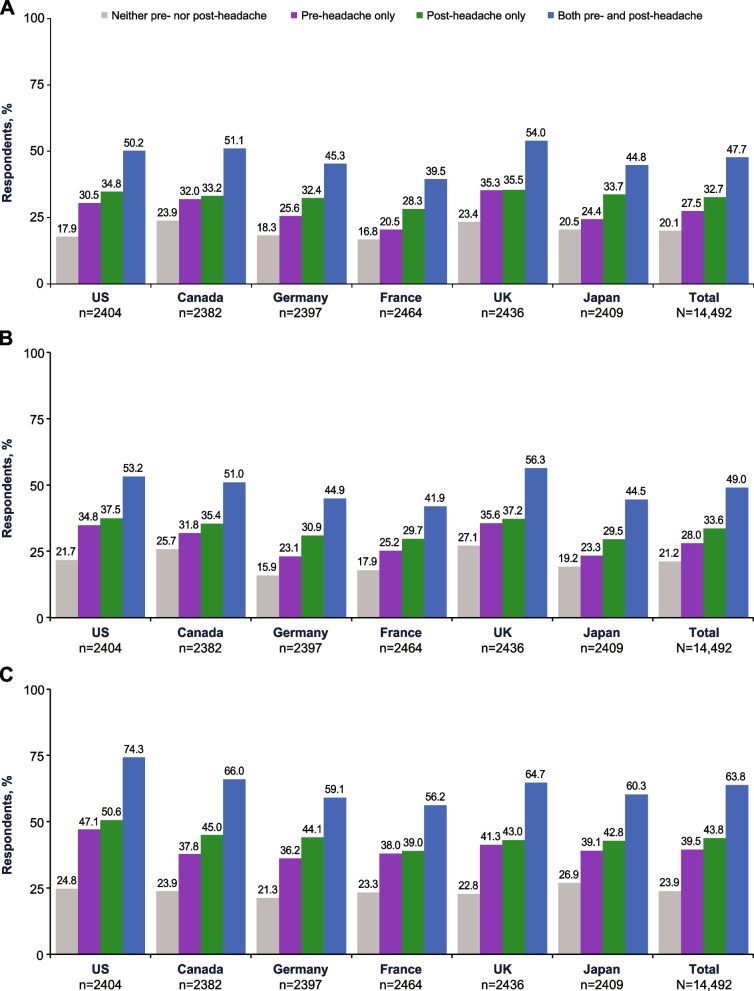
Percentages of Respondents With (**A**) Depression Symptoms,^a ^(**B**) Anxiety Symptoms,^a ^and (**C**) Allodynia by Pre- and Post-Headache Symptom Experience.^b^ UK, United Kingdom; US, United States. ^a^ Presence of depression and anxiety symptoms over the last 2 weeks was based on responses to the Patient Health Questionnaire-4. ^b^ Presence of allodynia during migraine was based on the 12-item Allodynia Checklist

Among respondents with neither pre- nor post-headache symptoms, 20.1% endorsed clinically significant symptoms of depression, and 21.2% endorsed clinically significant symptoms of anxiety (Fig. 4A and B). Clinically significant symptoms of anxiety were endorsed by 43.5% and 46.2% of respondents with any pre-headache or any post-headache symptoms, respectively. Of respondents with pre-headache symptoms only, 27.5% reported depression symptoms, while 28.0% reported anxiety symptoms. Of those with post-headache symptoms only, 32.7% reported depression symptoms and 33.6% reported anxiety symptoms. Among respondents with both pre- and post-headache symptoms, 47.7% reported symptoms of depression, and 49.0% reported symptoms of anxiety. Based on models adjusted for covariates, those with pre-headache symptoms only, post-headache symptoms only, or both pre- and post-headache symptoms were, respectively, 1.36, 1.64, and 2.92 times more likely to have anxiety symptoms than those with neither pre- nor post-headache symptoms. Similarly, those with pre-headache symptoms only, post-headache symptoms only, or both pre- and post-headache symptoms were, respectively, 1.39, 1.73, and 2.94 times more likely to have depression symptoms than those with neither pre- nor post-headache symptoms.

#### ASC-12

Among respondents with neither pre- nor post-headache symptoms, 23.9% reported allodynia (Fig. 4C). Allodynia was reported by 57.5% and 60.2% of respondents with any pre-headache or any post-headache symptoms, respectively. Of those respondents with pre-headache symptoms only, 39.5% reported allodynia, and among those with post-headache symptoms only, 43.8% reported allodynia. Of respondents with both pre- and post-headache symptoms, 63.8% reported the presence of allodynia.

#### Functional disability due to pre-headache, headache, and/or post-headache symptoms

**Fig. 5:**
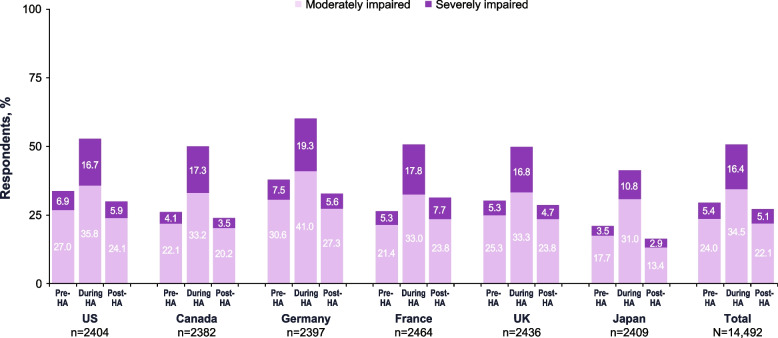
Percentages of Respondents With Moderate or Severe Functional Impairment/Activity Limitations During the Pre-Headache, Headache, and Post-Headache Phases.^a^ HA, headache. ^a^ “Before, during, and after your severe headache or migraine attacks, on average, how would you rate your performance of daily activities?”

During the headache phase, 51.0% of respondents reported moderate-to-severe functional and activity limitations (Fig. 5). During the pre-headache phase, 29.5% of respondents reported moderate-to-severe activity limitations. In the post-headache phase, 27.2% of respondents reported moderate-to-severe impairment.

#### mTOQ-4

**Fig. 6:**
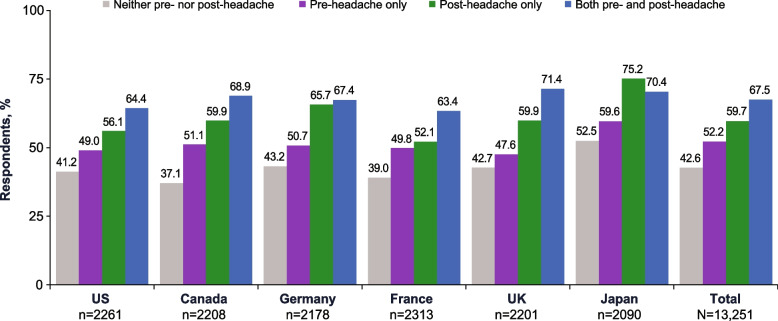
Percentages of Respondents With Migraine Who Used Acute Medication and Reported Poor to Very Poor Acute Treatment Optimization by Pre- and Post-Headache Symptom Experience.^a^ UK, United Kingdom; US, United States. ^a^ Based on scores from the Migraine Treatment Optimization Questionnaire

Among acute medication users with pre-headache symptoms only, 52.2% reported poor to very poor acute treatment optimization (Fig. 6). Among acute medication users with any pre-headache symptoms, 55.5% reported poor acute treatment optimization and 8.2% reported very poor acute treatment optimization. Among respondents who used acute medication and had post-headache symptoms only, 59.7% reported poor to very poor acute treatment optimization. Among respondents who used acute medication and had any post-headache symptoms, 57.3% reported poor treatment optimization and 8.9% reported very poor acute treatment optimization. Of those who used acute medication and had both pre- and post-headache symptoms, 67.5% of respondents reported poor to very poor acute treatment optimization. Based on modeling, those with pre-headache symptoms only, post-headache symptoms only, or both pre- and post-headache symptoms were, respectively, 1.32, 1.96, and 2.42 times more likely to have very poor to poor acute treatment optimization than those with neither symptoms.

## Discussion

Migraine is a multiphase, symptom-complex, chronic disease with episodic manifestations. This analysis of CaMEO-I data demonstrates the high rates of pre- and/or post-headache symptoms reported by people with migraine within the global population of people with migraine. Our analysis focused on the symptoms and associated burden of pre-headache and post-headache symptoms. At least 74% of respondents in each country reported experiencing symptoms in at least 1 of those non-headache phases. Respondents with both pre- and post-headache symptoms reported higher rates of migraine-related burden compared with those with pre-headache symptoms only, post-headache symptoms only, or neither pre- nor post-headache symptoms. Moderate-to-severe MIDAS scores were reported by more than 3.5 times the respondents who experienced both pre- and post-headache symptoms compared with those who experienced neither in each country. Functional impairment and activity limitations due to symptoms were greatest during the headache phase, but moderate-to-severe impairment was substantial during both the pre- and post-headache phases. Although differences were noted among countries, the patterns of findings were generally similar across the 6 countries.

Pre-headache symptoms have been the focus of numerous studies [1, 6, 8–10, 12, 13]. In a cross-sectional study, higher levels of headache frequency were associated with a higher frequency and number of pre-headache symptoms [6]. In an electronic diary study, participants assessed the likelihood of a migraine attack based on their premonitory symptoms and, as their certainty of prediction increased, so did the probability of a migraine attack [9]. The findings from the present study further support the literature, as a higher percentage of respondents who experienced only pre-headache symptoms were in higher MHD categories than those who did not have pre- or post-headache symptoms.

Prior studies of post-headache symptoms concentrated on the prevalence of these symptoms [4, 20]. The MiCOAS qualitative interviews found that 67.5% of respondents reported fatigue, 30.0% reported increased appetite, and 15.0% reported dull head pain as post-headache symptoms [10]. However, findings from an additional analysis of the MiCOAS study demonstrated that some symptoms may become less noticeable or bothersome because of increasing head pain [12]. The MiCOAS qualitative interview showed the wide range of symptoms reported across migraine phases, including symptoms such as cognitive impairments and emotional and psychological symptoms and states [11]. The postdrome phase can also cause significant, persistent disability and may be the least understood phase of migraine [3]. In an electronic diary study, disability associated with post-headache symptoms was considered to contribute to the burden experienced by those with migraine; however, a comparison with those who did not experience post-headache symptoms was not provided [4]. In the current study, the functional impairment and activity limitations by phase were greater among those who reported only post-headache symptoms compared with those who did not report pre- or post-headache symptoms.

Poor attack control, a higher frequency of attacks, and higher interictal burden have been linked to an increased risk of chronification [21, 22]. In the current study, those who experienced both pre- and post-headache symptoms were more likely to report 4–7, 8–14, and ≥ 15 MHDs than were those without pre- or post-headache symptoms. Furthermore, the present study suggests that the presence of prodromal and postdromal symptoms are associated with attacks that are more severe and disabling. Although the biology of the prodromal and postdromal phases is still being investigated, it appears that hypothalamic activity and persistent brain stem activation, respectively, play a role in the pathophysiology of migraine [23]. Emerging evidence from the PRODROME trial suggests that treating during the prodrome with ubrogepant can prevent the onset of headache and also reduce the duration of prodromal symptoms [24]. In the absence of treatment, the presence of prodrome is associated with greater disability. If treatment during the prodrome reduces the frequency of headache and shortens the duration of functional impairment, this emerging treatment paradigm could improve outcomes.

This study is limited by the self-reported nature of the data and the possibility of selection bias due to the use of an online survey platform. Also, we did not include all possible symptoms in our pre-populated survey; therefore, some symptoms may not have been captured, leading to underestimating occurrence. In particular, the presence of aura was not included in the analysis. Additionally, a link between headache frequency and pre- and post-headache symptoms was not evaluated in this analysis; however, the burden among those with pre- and post-headache symptoms (in particular, monthly headache categories) is of interest. Further limitations include the absence of data analyzed regarding medication use and particular symptoms among those who experienced pre- and post-headache phases. These limitations are countered by the strengths of this study, including the large sample size across multiple countries, quality checks to ensure the validity of surveys, and the use of validated questionnaires to assess migraine-related burden.

Clarifying our understanding of pre-headache symptoms could improve our ability to study the underlying mechanisms of attack onset and may lead to earlier treatment during the prodrome, with the intention of shortening the prodrome, preventing headaches, and improving function. Given the complex nature of migraine attacks, further studies of person-level and population-level links to pre-headache and post-headache symptoms are required. Furthermore, the suboptimal treatment demonstrated among individuals with pre-and post-headache symptoms in this study suggests that future research may be warranted to explore pre- and post-headache phases as potential targets for treatment.

## Conclusions

Pre-headache symptoms were reported by 66.9% of respondents, while post-headache symptoms were reported by 60.2% of respondents. Across countries, monthly headache days, rates of moderate-to-severe migraine-related disability, anxiety symptoms, depression symptoms, and allodynia were greatest among those who experienced both pre- and post-headache symptoms. Moderate-to-severe activity limitations were reported in 16–38% of people during the pre- and post-headache phases.

## Data Availability

AbbVie is committed to responsible data sharing regarding the clinical trials we sponsor. This includes access to anonymized, individual, and trial-level data (analysis data sets), as well as other information (eg, protocols, clinical study reports, or analysis plans), as long as the trials are not part of an ongoing or planned regulatory submission. This includes requests for clinical trial data for unlicensed products and indications. These clinical trial data can be requested by any qualified researchers who engage in rigorous, independent, scientific research, and will be provided following review and approval of a research proposal, Statistical Analysis Plan (SAP), and execution of a Data Sharing Agreement (DSA). Data requests can be submitted at any time after approval in the US and Europe and after acceptance of this manuscript for publication. The data will be accessible for 12 months, with possible extensions considered. For more information on the process or to submit a request, visit the following link: https://vivli.org/ourmember/abbvie/, and then select “Home.”

## Abbreviations

ASC-12: Allodynia Symptom Checklist
CaMEO-I: Chronic Migraine Epidemiology and Outcomes Study – International
ICHD-3: *International Classification of Headache Disorders*, 3rd edition
MHD: Monthly headache day
MIDAS: Migraine Disability Assessment
mTOQ: Migraine Treatment Optimization Questionnaire
PHQ-4: Patient Health Questionnaire
